# *Stenotrophomonas maltophilia* Outbreak in an ICU: Investigation of Possible Routes of Transmission and Implementation of Infection Control Measures

**DOI:** 10.3390/pathogens13050369

**Published:** 2024-04-29

**Authors:** Maria Luisa Cristina, Marina Sartini, Gianluca Ottria, Elisa Schinca, Giulia Adriano, Leonello Innocenti, Marco Lattuada, Stefania Tigano, David Usiglio, Filippo Del Puente

**Affiliations:** 1 Operating Unit Hospital Hygiene, Galliera Hospital, 16128 Genoa, Italy; maria.luisa.cristina@galliera.it (M.L.C.); gianluca.ottria@unige.it (G.O.); elisa.schinca@unige.it (E.S.); 2 Department of Health Sciences, University of Genoa, 16126 Genoa, Italy; 3 Hospital Infection Control Committee, Galliera Hospital, 16128 Genoa, Italy; giulia.adriano@galliera.it; 4 Department of Laboratory and Microbiological Analysis, Galliera Hospital, 16128 Genoa, Italy; leonello.innocenti@galliera.it (L.I.); david.usiglio@galliera.it (D.U.); 5 Anaesthesia and Intensive Care Unit, E.O. Ospedali Galliera, 16128 Genoa, Italy; marco.lattuada@galliera.it; 6 Department of Infectious Diseases, Galliera Hospital, 16128 Genoa, Italy; stefania.tigano@galliera.it (S.T.); filippo.del.puente@galliera.it (F.D.P.)

**Keywords:** *S. maltophilia*, outbreak, ICU, MLST

## Abstract

*Stenotrophomonas maltophilia*, a non-fermentative, ubiquitous, gram-negative aerobic bacterium, is associated with high mortality rates, particularly in immunocompromised or debilitated patients. The prevalence rate of ICU-acquired pneumonia episodes caused by this microorganism has been found to be 2%. *S. maltophilia* has been identified as one of the top 10 microorganisms responsible for such infections in EU/EEA countries. This study describes an outbreak of *S. maltophilia* in an intensive care unit of a hospital in northern Italy. This includes an epidemiological investigation of the cases, the environmental microbiological controls carried out, a comparison of the strains by multilocus sequence typing (MLST), and the measures taken to prevent and control the outbreak. Among the seven clinical isolates of *S. maltophilia* analyzed herein, six demonstrated susceptibilities to trimethoprim–sulfamethoxazole. Conversely, one isolate of *S. maltophilia* exhibited resistance to first-line antibiotics. ST was found to be identical for six patients (ST 4), as well as in the environmental feedback on the trolley of Box 2. The analysis of the temporal and spatial progression of the outbreak has suggested that the transmission of *S. maltophilia* may have occurred through cross-transmission during care practices.

## 1. Introduction

*Stenotrophomonas maltophilia*, a non-fermentative, ubiquitous, gram-negative aerobic bacterium, is frequently associated with nosocomial infections [1,2]. In these instances, the bacterium is associated with high mortality rates, particularly in immunocompromised or debilitated patients. Several characteristics of this pathogen contribute to the significant challenges it poses in the nosocomial setting [3,4,5]. A major challenge posed by *S. maltophilia* is its notable resistance to antibiotics [3], which is attributed to the production of inducible beta-lactamases that diminish the effectiveness of beta-lactams, including monobactams and carbapenems [6]. Moreover, acetyl-transferases diminish the efficacy of aminoglycosides, while the production of efflux pumps ensures resistance to diverse classes of antibiotics, such as fluoroquinolones [7]. Its ability to adhere to materials is facilitated by a positively charged surface and fimbriae, enhancing adhesiveness. Additionally, it demonstrates a capacity to form biofilms [8,9].

While *S. maltophilia* is ubiquitous, its pathogenic role is predominantly associated with nosocomial infections, prevalent in individuals with risk factors associated with a high degree of medicalization [4,10,11,12]. Such factors include, as with many other HAIs, admission to an ICU, neoplastic disease, CF, neutropenia, mechanical ventilation, presence of a central venous catheter, recent surgery, HIV infection, and prior treatment with broad-spectrum antimicrobials [2,4,13,14].

The clinical manifestations of *S. maltophilia* infection primarily occur in two forms: nosocomial pneumonia (often associated with mechanical ventilation) and sepsis (frequently linked to the presence of a central venous catheter) [15,16]. Pulmonary infections should be distinguished from colonization, and their course is akin to that of nosocomial pneumonia, except in cases of patients with hematological disorders who may develop a rapidly progressive syndrome associated with pulmonary hemorrhage [15].

Distinguishing between colonization and infection by *S. maltophilia* poses a significant clinical challenge [17]. The isolation of *S. maltophilia* from sterile fluids, such as blood, pleural or peritoneal fluid, and cerebrospinal fluid, unequivocally indicates an infection, necessitating immediate attention. Conversely, isolation from non-sterile fluids requires a comprehensive clinical investigation [18]. In instances of persistent uncertainty, the initiation of antibiotic therapy hinges on a prompt reassessment of treatment response, typically after a 48–72 h timeframe [3,19].

Specifically, differentiating between colonization and infection based on isolates from the respiratory tract poses a significant challenge due to *Stenotrophomonas*’ capacity to adhere to the upper respiratory tract [19]. In such cases, patients presenting with additional symptoms or signs of nosocomial pneumonia (such as diminished peripheral oxygenation, fever, leukocytosis, and emergence of new findings on the thorax CT scan) should be carefully considered for therapeutic intervention [20].

Infections caused by *S. maltophilia* lead to mortality rates ranging between 14% and 69% for bacteremia and between 23% and 77% for pneumonia [21]. The prevalence rate of ICU-acquired pneumonia episodes caused by this microorganism, based on the two Italian surveillance networks (GIVITI and SPIN-UTI), was found to be 2%. *S. maltophilia* was identified as one of the top 10 microorganisms responsible for such infections in EU/EEA countries [22].

The aim of this study is to describe an outbreak of *S. maltophilia* in an intensive care unit of a hospital in northern Italy. This includes an epidemiological investigation of the cases, the environmental microbiological controls carried out, a comparison of the strains by multilocus sequence typing (MLST), and the measures taken to prevent and control the outbreak.

## 2. Materials and Methods

Cases were defined as patients admitted to the Intensive Care Unit who tested positive for *S. maltophilia* through a bronchoaspiration culture or another method, such as a blood culture. This particular case series comprises a total of 7 patients, spanning from 13 August 2018 to 19 September 2018.

The study was conducted in a nationally renowned, highly specialized northern Italian hospital with a capacity of 458 beds (mainly in 3- and 4-bed rooms), more than 15,000 routine admissions per year, and over 8600 medical procedures in outpatient and day surgery settings. The ICU consists of an open space with 6 beds and 2 additional beds designated for patient isolation, referred to as Boxes 1 and 2.

The outbreak was identified by means of continuous integrated microbiological surveillance, starting from laboratory data (alert organism surveillance), following identification by the laboratory of an “epidemiological important microorganism”. Then, the dedicated software of the surveillance system Concerto (Dedalus Italia, Florence, Italy) automatically sent the data by e-mail to all the members of the Hospital Infections Committee, who then implemented the interventions deemed necessary, with particular regard to the application of isolation measures. A validated report was simultaneously sent through the Laboratory Information System to the hospital facility involved (in this case, the ICU).

The reconstruction of the infection spread took into account various factors, including the chronologically compatible hospitalization periods of patients, length of stay, administered therapies or diagnostic investigations in the same environments, and medical consultations by healthcare personnel from departments where infection cases were ascertained.

### 2.1. Environmental Investigation

Microbiological surveillance was initiated in response to the outbreak onset on 28 August and effectively started two days later.

The first monitoring campaign was conducted in the ICU, carrying out sampling on different surfaces, including bed heads, bed rails, door handles, light switches, intercoms, monitors, infusion pumps, manometers, stethoscopes, glucose meters, portable ultrasound scanners, disinfectants, physiological solutions, keyboards of staff computers and telephones, bedside trolleys, sinks (washbasins, soap dispensers taps, shelves, and sink tops), and patient care products. The environmental samples were obtained using sterile swabs with a neutralizing rinse solution (Liofilchem srl, Roseto degli Abruzzi (TE), Italy).

Despite point-of-use water filters (with pore size 0.2 mm) being installed on every tap in the ICU, water sampling was carried out.

Tap water was collected into sterile 500 mL containers and filtered onto a membrane with a 0.45 micrometer pore size (Millipore, Bedford, MA, USA). The membrane was imprinted onto McConkey agar (Liofilchem srl, Roseto degli Abruzzi (TE), Italy). Any non-lactose fermenting colonies were identified by MALDI system (bioMérieux, Marcy l’Etoile, France) analysis and, if *S. maltophilia* was found, sent for typing by MLST.

The bronchoscopes used in the ICU were all single-use and disposable, so they were not sampled.

Microbiological control, performed on a random basis, confirmed the absence of microbial growth and, thus, *S. maltophilia*. Subsequently, the investigation expanded to include the evaluation of Operating Room 1 and the CT radiodiagnostic room, where colonized patients had been present.

Environmental monitoring was subsequently repeated on 10 and 12 September, prompted by the emergence of two additional cases.

A total of 153 environmental samples were carried out.

### 2.2. Microbiological Analysis and Molecular Typing of Clinical and Environmental Samples

Clinical samples were obtained from bronchoaspiration. Bronchoaspiration was performed by aspiration of bronchial secretions after instilling a variable amount (usually 20 mL) of saline in a sterile manner. In cases of surveillance bronchoaspiration (thus without pathological findings on imaging), this was performed in the bronchial secretions present; in cases of abnormal findings on imaging, bronchoaspiration was performed in the area of interest. All *S. maltophilia* isolates (clinical and environmental samples) were identified by means of the MALDI system (bioMérieux, Marcy l’Etoile, France) and characterized for their antibiotic sensitivity using Phoenix 100 (Becton Dickinson, Franklin Lakes, NJ, USA).

The isolates underwent molecular typing by means of the multilocus sequence typing (MLST) technique, which involves amplifying and sequencing seven housekeeping genes (atpD, gapA, guaA, mutM, nuoD, ppsA, and recA) and, through comparison with the data available in an online databank, enables a specific ST (Sequence Type) to be assigned to each isolate. The amplification PCR protocol (https://pubmlst.org/organisms/stenotrophomonas-maltophilia/primers, accessed on 5 February 2024) prescribes an initial activation of the Taq-DNA-Polymerase for 9 min at 95 °C, followed by 30 cycles of 20 s denaturation at 94 °C, annealing for 1 min at the appropriate annealing temperature, and extension for 50 sec at 72 °C.

The sequencing reaction was performed with 20 ng DNA and the BigDye Terminator Ready Reaction Mix (v1.1, Applied Biosystems, Foster, CA, USA). Cycle sequencing with standard conditions was used for primers with Ta > 60 °C. In the case of lower Ta, denaturation for 10 sec at 96 °C was followed by annealing at Ta for 10 sec and subsequent elongation for 4 min at 60 °C.

### 2.3. Institutional Review Board

The study was conducted according to the guidelines of the Declaration of Helsinki. Ethical review and approval were waived for this study because all analyzed data were extracted anonymously by the laboratory, and the study did not involve humans or animals. Personal and biological data were not used in the present study, and given the legal wording of the Italian law, a full review board opinion was not necessary since it does not fall within the cases involved by the “European Clinical Trials Regulation”, not being a clinical trial or a study in which biological samples were collected and not involving a clinical intervention (https://www.istitutoitalianoprivacy.it/2024/01/10/proposta-di-riforma-per-la-privacy-e-la-ricerca-scientifica-tavolo-salute-di-state-of-privacy/; https://www.aifa.gov.it/documents/20142/516919/111.88758.1186138046156a0be.pdf/fd749d81-e385-0ec1-2178-5954d97484a3; https://www.aifa.gov.it/en/regolamento-europeo-sperimentazioni-cliniche, accessed on 5 February 2024).

## 3. Results

Among the seven clinical isolates of *S. maltophilia* analyzed, six demonstrated susceptibilities to trimethoprim–sulfamethoxazole (ST 4, patients 1–4, 6, and 7). Conversely, one isolate of *S. maltophilia* exhibited resistance to first-line antibiotics, prompting a supplementary sensitivity analysis. This additional analysis revealed that the strain was susceptible only to colistin (ST 208, patient 5).

### 3.1. Outbreak Description

During August 2018, an outbreak occurred involving seven patients who were admitted to the ICU. In the previous year, there were no other registered cases of *S. maltophilia* nosocomial pneumonia or sepsis in the ICU. The last colonization had occurred 13 months before the onset of the outbreak.

During this period, 58 bronchoaspirations were conducted on a total of 34 patients, using disposable bronchoscopes. To comprehend the progression of *S. maltophilia* colonization and its subsequent impact, patient histories and clinical treatments were thoroughly evaluated.

**Figure 1:**
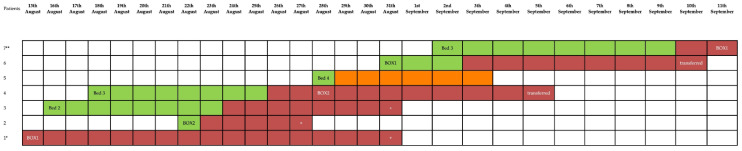
Distribution of *Stenotrophomonas maltophilia*-positive patients as a function of time and the occupancy of beds: “+” death. Patient 1* entered the ICU on 23 July and became positive on 13 August. Patient 7** continued his hospitalization in the ICU until 26 September.

The outbreak, comprising a total of seven patients, originated from the index case (Patient 1) admitted to the ICU on 6 August. Figure 1 illustrates the temporal distribution of patients testing positive for *S. maltophilia*, while Table 1 illustrates the clinical condition of patients at ICU admission.

The index case, a 47-year-old man, was admitted to the hospital due to acute necrotizing pancreatitis and subsequently transferred to the ICU (Box 1) on 23 July. Treatment included broad-spectrum antimicrobials (piperacillin/tazobactam, linezolid, and caspofungin). A CT scan on 24 July revealed pleural effusion without interstitial infiltrates. Despite antimicrobial therapy, procalcitonin values continued to rise. A subsequent thoracic X-ray on 12 August identified new, patchy infiltrates. On 13 August, *S. maltophilia* was isolated from the culture obtained through bronchoaspiration (BAS). Despite antimicrobial therapy targeting *S. maltophilia,* the patient died on 31 August.

Patient 2, admitted to the ICU on 22 August (Box 2), tested positive for *S. maltophilia* on 23 August. The patient passed away on 27 August following a general worsening of the clinical condition due to *S. maltophilia* infection. Pre-ICU consultations with operators in the sub-ICU suggest a plausible contraction from patient 1.

Patient 3, initially admitted to general surgery, was transferred to the Intensive Care Unit (ICU) on 16 August (Bed 2). On 24 August, a bronchoaspiration procedure was conducted, which resulted in the detection of *S. maltophilia*. Unfortunately, the patient passed away on 31 August due to other complications that arose during their ICU stay. It was therefore determined that the patient was colonized, but not infected, by *S. maltophilia*. The likely source of contraction was Patient 2.

Patient 4 was admitted to the ICU on 18 August (Bed 3) and tested positive for *S. maltophilia* on 26 August. Treatment started on 28 August, and the patient was subsequently transferred to Box 2 on the same day. There was a potential direct contraction of the infection from Patient 3, considering their adjacent beds. On 5 September, the patient was transferred to another ward.

Patient 5 was admitted to the ICU on 28 August (Bed 4) and tested positive for *S. maltophilia* the day after, suggesting that a previous colonization occurred during hospitalization in another department. Treatment for *S. maltophilia* was started immediately. Discharge from the ICU occurred on 4 September. Initially, her isolate was considered part of the outbreak, but subsequent multilocus sequence typing revealed a different sequence type (ST) compared to other strains.

Admitted on 31 August (Box 1), Patient 6 tested positive for *S. maltophilia* upon a bronchoaspiration culture on 3 September. Isolation of the pathogen was considered as a colonization. He may have contracted the colonization directly from Patient No. 4, given the concomitant admission of the two patients to adjacent beds for several days. The patient was discharged from the ICU on 10 September.

Patient 7 was admitted to the ICU (Bed 3) on 2 September, tested positive on 10 September, and was then moved to Box 1. He was later discharged to the sub-ICU on 26 September. It is most likely that the colonization was contracted from Patient 6, the only colonized patient remaining during the days prior. No treatment against *S. maltophilia* was started in this case. This case was the last of the outbreak; no other colonization or infection was registered until 2023.

**Figure 2:**
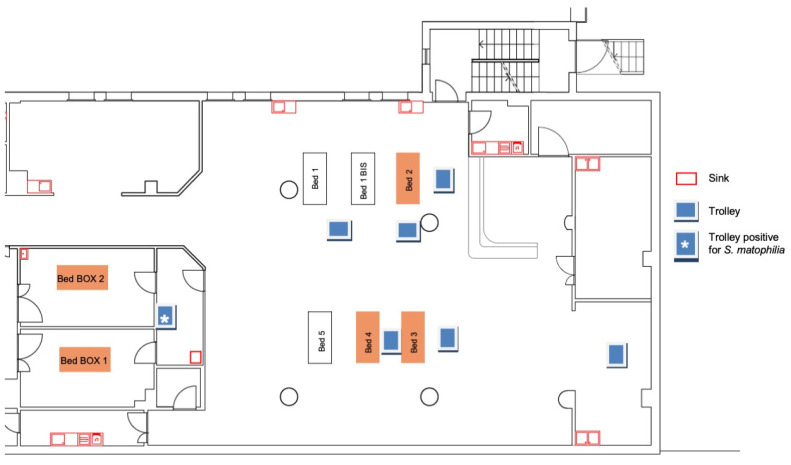
ICU beds occupied by patients positive for *S. maltophilia* during the outbreak.

Figure 2 displays the beds that were occupied by the patients who tested positive for *S. maltophilia*.

### 3.2. Results of Environmental Investigation

The first round of sampling (30 August), consisting of 59 samples, revealed only one sample positive for *S. maltophilia* (15 CFU/cm^2^) on the surface of the trolley in Box 2, where Patients 2 and 4 were hospitalized, while Patients 1, 3, and 5 had not yet been transferred there until that moment. Furthermore, Patient 4 had been colonized by Stenotrophomonas before his transfer to Box 2.

The second series of samplings was conducted on 10 September, with a total of 49 samples, all with negative results.

Finally, the third series of samplings was carried out on 12 September, totaling 45 samples, all of which also tested negative.

### 3.3. Biomolecular Results

ST was found to be identical for Patients 1, 2, 3, 4, 6, and 7 (ST 4), as well as in the environmental feedback on the trolley of Box 2. However, Patient 5 exhibited a different ST (ST 208) (Table 2). These data confirmed that the transmission occurred through the operators, and the isolation carried out on the trolley confirmed a deficiency in the sanitization procedures, potentially exacerbated by a temporary personnel shortage which occurred in the period affected by the epidemic.

As described in the outbreak description, the hypothesized transmission is, therefore, as follows: from Box 1 to Box 2, then to Bed 2, to Bed 3, then to Box 1, and finally to Bed 3 again. From the analysis of the routes, movements, and locations, it does not appear that a single patient unit or ward area common to all cases was involved.

According to the PubMLST database, ST 4 has been isolated in clinical samples from different European countries, including Italy, and from Thailand, while ST 208 has been found in blood and sputum from the USA and Thailand, respectively [23].

### 3.4. Infection Control Measures

The response entailed the implementation of heightened infection control measures, including enhanced standard procedures; daily 2% chlorhexidine patient washing; and twice-daily unit sanitization utilizing, whenever possible, sodium dichloroisocyanurate at 1000 ppm available chlorine or, alternatively, 70% alcohol.

Checklists were implemented for monitoring, and signed confirmations ensured compliance. Materials in proximity to colonized patients were removed and sent for additional microbiological investigation. Early surveillance bronchoalveolar lavages were conducted for all patients during the study period. Staff were reminded of the prohibition of jewelry and nail polish, and dedicated nursing staff were assigned to specific zones.

An AUDIT was conducted in the department to verify the adequacy, adherence, and comprehension of protocols and operating instructions by healthcare workers. The standard precautions and additional precautions for contact in cases colonized by Stenotrophomonas were applied with particular attention (chlorhexidine, washing the patient unit 2 times a day, and checking the checklist).

Surveillance bronchoaspirations were brought forward, cohorting was implemented where possible with functional isolation, and hand hygiene practice was stressed. A color control infection (color code) was launched which assigned a color to each type of physical and functional isolation.

A nurse was immediately dedicated to the sole control of colonized patients. Daily infectious disease assessments were requested to evaluate the antibiotic therapy of all patients.

Upon the detection of trolley contamination in Box 2 through environmental monitoring, heightened measures were promptly emphasized to mitigate the spread of the microorganism.

## 4. Discussion

The objective of the present study is to describe an outbreak that occurred in an ICU and to investigate the probable transmission routes of the microorganism by employing the study of the allelic profiles of the isolated *S. maltophilia* strains. We also investigate the presence of possible reservoirs within the ICU and, more generally, of the diagnostic routes taken by patients before the detection of colonization by *S. maltophilia*. Additionally, we describe the strategies employed in managing the outbreak and its outcome.

The analysis of the temporal and spatial progression of the outbreak has suggested that the transmission of *S. maltophilia* may have occurred through cross-transmission during care practices, even though this conclusion was not supported by the microbiological analysis of the operators’ hands. Contaminated hands of healthcare providers are a primary source of pathogenic spread. Proper hand hygiene decreases the proliferation of microorganisms, thus reducing infection risk. According to the Centers for Disease Control and Prevention (CDC), hand hygiene is the single most important practice in the reduction in the transmission of infection in the healthcare setting [24].

To substantiate this claim, it is important to highlight that the outbreak was supported by the same allelic profile in six out of seven cases, and that it took place during the summer period, coinciding with a decrease in the number of ICU staff and resulting in a reduction in staff–patient interactions. This could potentially explain a decline in compliance with outbreak prevention practices, including the sanitization of surfaces in the patients’ hospital area [25].

In response to the emergence of the initial cases of *S. maltophilia* infection, measures were taken to contain the spread of the microorganism. These measures included the implementation of audits; an analysis of care processes; microbiological analysis of the environment and care pathway; and, lastly, the biomolecular analysis of bacterial strains. Following the results of environmental monitoring, the implementation of additional containment measures led to a decrease in the number of colonized patients in the days following the initial cases, ultimately resolving the outbreak in a short period of time. It is also important to highlight that the monitoring efforts ensured that the case supported by a different ST than the others did not spread further, remaining an isolated case within the outbreak. The implementation of the environmental monitoring measures helped to support the adherence of healthcare workers to the hygiene approach, as evidenced by the microbiological results obtained during the additional environmental monitoring performed in the later stages of the outbreak (10 and 12 September).

The implementation of these measures and the multidisciplinary approach successfully prevented further incidents of Stenotrophomonas colonization in the subsequent five years, underscoring the effectiveness of a comprehensive and proactive approach to infection control [26,27,28]. The outbreak, in fact, subsequently ended with no new cases or colonization occurring after Patient 7 during his stay, which continued in the ICU until 26 September, the date of his transfer to the sub-ICU, where he maintained colonization-related precautions. No other cases of infection or colonization occurred thereafter in the ICU until 2023. Previous investigations have examined outbreaks of *S. maltophilia* in the ICU. In our study, the presence of *S. maltophilia* was documented in a single location (trolley in Box 2). The detection of *S. maltophilia* in limited environmental settings aligns with findings from other studies [29,30,31]. In our investigation, the presence of *S. maltophilia* was detected in seven patients. The observation of a relatively small number of colonized patients is consistent with other outbreak scenarios [30,32], but its importance should be weighted with the high mortality associated with infections caused by this pathogen [33,34].

On the other hand, our cohort stands apart from other outbreak scenarios due to several factors: (1) All patients with *S. maltophilia* infection developed respiratory colonization with or without nosocomial pneumonia, with no cases of bacteremia; (2) no diagnostic or therapeutic material was found to be contaminated [35,36]; (3) a single principal ST was found to be the culprit of the outbreak [37]; and (4) our investigation did not find evidence of polymicrobial colonization or infection from bronchoaspiration samples [38].

A notable advantage of our study lies in the ability to type each *S. maltophilia* strain using the same methodology.

In this study, the MLST technique was employed, which, as indicated by the scientific literature [39,40], has been shown to be a reliable tool for tracking the source of infection and the distribution of pathogens isolated from hospitalized patients. This technique yields consistent epidemiological data, and results from various laboratories can be compared due to the easy accessibility of international databases.

This allowed us to comprehensively map the outbreak’s spatial and temporal development, providing valuable insights. Such an opportunity is not always available in biological research [30]. Furthermore, we must highlight the relatively rapid containment of the outbreak, which was resolved within approximately one month. This stands in contrast to other literature reports that have documented outbreaks persisting for several months [34].

### Limitation

The environmental study was not extended to the evaluation of hand washing, and therefore, no culture evaluation of the hands of healthcare workers was carried out, since the decision was to prioritize the microbiological surveillance of the clinical pathways and environment. This limitation does not allow us to conclude with absolute certainty that the transmission of *S. maltophilia* occurred via health care workers; however, the finding of only one environmental isolation at the beginning of the investigations suggests an operator-dependent transmission. Unfortunately, the absence of a timely decision by hospital management and the compliance of operators did not make it possible to highlight this finding.

## Figures and Tables

**Table 1:** Clinical and microbiological data of patients.

Patients	Age (Years)/Sex	Transferred from Other Healthcare Facilities	Admission Diagnosis	Admission Ward	Hospital Stay (ICU) before First Isolation (Days)	Isolate Site	Other Isolation from the Same Site	Outcome	Surgical Procedures
1	47/M	No	Acute necrotizing pancreatitis	Sub-ICU	23	Bronchoaspiration	no	Died	Surgical intervention and surgical redo
2	78/M	No	Acute respiratory insufficiency	Sub-ICU	30	Bronchoaspiration	no	Died	-
3	58/M	No	Acute pancreatitis post-surgery	General surgery	7	Bronchoaspiration	no	Died	Cholecystectomy, surgical treatment for peritonitis
4	70/M	Yes	Aortic dissection	Vascular surgery	25	Bronchoaspiration	no	Transferred	Surgical repair of aortic aneurism
5	50/F	No	Sepsis and respiratory insufficiency	Neurosurgery	49	Bronchoaspiration	no	Transferred	Meningioma excision
6	56/M	No	Acute respiratory distress syndrome	Emergency Department	0	Bronchoaspiration	no	Transferred	-
7	63/M	No	Acute respiratory distress syndrome	Emergency Department	0	Bronchoaspiration	no	Transferred	-

F: female; M: male; Sub-ICU: sub-Intensive Care Unit; ICU: Intensive Care Unit.

**Table 2:** *Stenotrophomonas maltophilia* allelic profiles (clinical samples related to patients and environmental sample).

ALLELIC PROFILES									
*atpD*	*gapA*	*guaA*	*mutM*	*nuoD*	*ppsA*	*recA*	ST	Patients	Environmental Sample
4	76	155	5	70	84	9	208	5	-
1	4	7	7	28	19	6	4	1, 2, 3, 4, 6, 7	Trolley Box 2

## Data Availability

The data presented in this study are available upon request from the corresponding author. The data are not publicly available due to internal regulations.
